# HDC-Net: A hierarchical dilation convolutional network for retinal vessel segmentation

**DOI:** 10.1371/journal.pone.0257013

**Published:** 2021-09-07

**Authors:** Xiaolong Hu, Liejun Wang, Shuli Cheng, Yongming Li

**Affiliations:** College of Information Science and Engineering, Xinjiang University, Urumqi, China; University of Engineering & Technology, Taxila, PAKISTAN

## Abstract

The cardinal symptoms of some ophthalmic diseases observed through exceptional retinal blood vessels, such as retinal vein occlusion, diabetic retinopathy, etc. The advanced deep learning models used to obtain morphological and structural information of blood vessels automatically are conducive to the early treatment and initiative prevention of ophthalmic diseases. In our work, we propose a hierarchical dilation convolutional network (HDC-Net) to extract retinal vessels in a pixel-to-pixel manner. It utilizes the hierarchical dilation convolution (HDC) module to capture the fragile retinal blood vessels usually neglected by other methods. An improved residual dual efficient channel attention (RDECA) module can infer more delicate channel information to reinforce the discriminative capability of the model. The structured Dropblock can help our HDC-Net model to solve the network overfitting effectively. From a holistic perspective, the segmentation results obtained by HDC-Net are superior to other deep learning methods on three acknowledged datasets (DRIVE, CHASE-DB1, STARE), the sensitivity, specificity, accuracy, f1-score and AUC score are {0.8252, 0.9829, 0.9692, 0.8239, 0.9871}, {0.8227, 0.9853, 0.9745, 0.8113, 0.9884}, and {0.8369, 0.9866, 0.9751, 0.8385, 0.9913}, respectively. It surpasses most other advanced retinal vessel segmentation models. Qualitative and quantitative analysis demonstrates that HDC-Net can fulfill the task of retinal vessel segmentation efficiently and accurately.

## Introduction

The study found that the number of patients with retinopathy increases with the advent of an aging population. There are many reasons for retinopathy, such as diabetes, nephritis, anemia, influenza, which may cause fundus diseases. The clinical symptoms of retinopathy are mainly manifest in changes in the length, width, curvature, and angle of the retinal blood vessels [1]. For instance, diabetic retinopathy [2] is associate with swelling of the blood vessels, and hypertensive retinopathy [3] is accompanied by increased retinal vessel curvature and narrowing of blood vessels. Although retinopathy can be observed in many ways, the most critical characteristic is the variation of retinal blood vessels.

To enable sufferers to receive reasonable treatment, ophthalmologists usually diagnose related diseases by observing the morphological features of the abnormal blood vessels. Therefore, to observe exceptional blood vessels more intuitively, it is most crucial to analyze blood vessels’ structure from fundus images accurately. However, it is not easy for researchers to obtain clear segmentation images. Researchers will be affected by the different colors, contrasts, foregrounds, and backgrounds of fundus images when extracting retinal blood vessels. At the same time, the fundus image is easily affected by uneven illumination and noise [4], making the task of blood vessel extraction quite challenging. Some experienced experts will be disturbed by retinal disease and low contrast images, making the artificial extraction of retinal vessels error-prone and time-consuming. Therefore, a high-quality fundus image plays a critical part in the early condition analysis and subsequent treatment of ophthalmic diseases. In addition, retinal blood vessel segmentation can also be used in partial cell ophthalmology research and is a necessary condition for pre-research treatment.

In recent decades, deep learning enthusiasts have found many feasible strategies for obtaining a more precise retinal vessel segmentation map. Based on little earlier information, conventional retinal vessel extraction strategies can further be divided into matched-filtered (MF) methods [5–7], mathematical morphology methods [8–10], model-based methods [11–13], and vessel tracking methods [14, 15]. These strategies utilize hand-crafted features such as shapes, spatial areas, and edges for precise retinal vessel extraction.

With the fast advancement of deep learning, advanced architecture and modules have been proposed and applied to different fields of computer vision, such as image segmentation [16], speech recognition, text detection, and a series of tasks based on deep learning. The unique superiority of convolutional neural networks (CNNs) [17] is that they can adequately represent and learn the image features, so methods based on CNN’s often utilized in medical image classification tasks. In the diagnosis of some diseases, the primary mission is to segment and analyze the structure of cells in detail. The proposal of the U-Net [18] networks can get a clear image of cell structure from medical images so that they can analyze the condition further. Compared with traditional CNNs, U-Net proposed based on fully convolutional network (FCNs) [19] represent the learning feature information from rough to delicate. U-Net has accomplished an extraordinary victory within the field of medical image segmentation and has inspired other applications of U-shaped structures for retinal vessel segmentation. However, many U-Net variants are unable to detect the blood vessels in fundus images adequately. Consequently, we proposed HDC-Net based on U-Net, which can fully capture the features of blood vessels that are often ignored in fundus images. To whole up, the contributions of this article are summarized as follows: (1) we proposed a u-shaped structure that contains HDC modules, which detects vessel features of different scales by adjusting the receptive fields of the convolution kernel to obtain more accurate segmentation results. (2) The RDECA module was obtained by improving the efficient channel attention mechanism. We apply the RDECA module to the skip connection, focusing on channel information more conducive to segmentation and enhancing the model’s discriminant capacity.

In this paper, the second section is a brief literature review of the relevant network. In the third section, the architecture of HDC-Net and related modules are introduced in detail. The fourth section mainly introduces the related datasets and metrics. The fifth section presents experimental results and evaluates the model on two datasets. The sixth section gives conclusions and discussions.

## Related work

Image segmentation is a hot topic in deep learning, and the medical image is one of the critical research objects. Retinal blood vessel segmentation firstly locates and recognizes blood vessels and then segments them. With the innovation of deep learning, various intelligent algorithms were applied to obtain a more precise map of the vascular structure, among which researchers have highly praised the supervised methods. Supervised learning requires manually labeling the data to establish an optimal predictive model. Researchers input the processed image into an excellent prediction model to obtain the corresponding probability prediction map.

Fundus image datasets are susceptible to quality degradation due to noise and illumination during acquisition, so dataset pre-processing is a key step in image analysis. Datasets are augmented in various ways, such as random rotation, random flipping, color Jittering [20] and a host of other ways to increase the number of images. As the target vessels and background are not easily distinguishable in fundus images, it is common to use contrast limited adaptive histogram equalization (CLAHE) to improve image contrast. In addition, some scholars have continued to innovate on this basis; for example, Li et al. proposed to combine CLAHE with the discrete wavelet transform [21] to preserve good image detail and suppress noise, Khursheed Aurangzeb et al. proposed to tune the CLAHE parameters using particle swarm optimization algorithm [22] to improve the contrast of the images of green channel.

**Fig 1:**
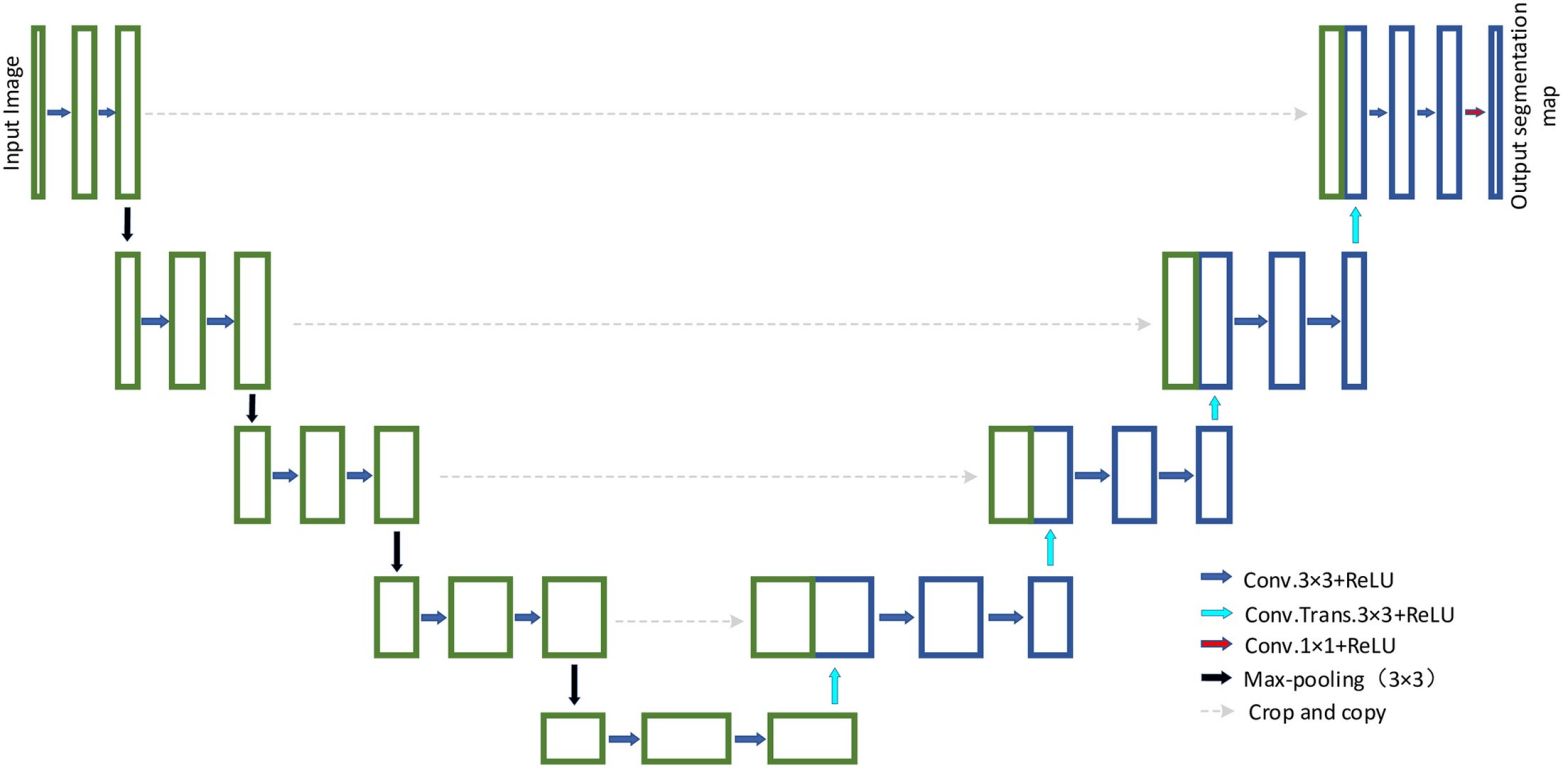
The main architecture of the initial version of U-Net.

U-Net has an important position within the field of medical imaging analysis. As shown in Fig (1), the leading architecture of U-Net is mainly composed of a convolutional coding unit and decoding unit. The basic convolution operation is performed, followed by ReLU activation in the encoding and decoding unit. The 2×2 max-pooling operation is used for down-sampling in the encoding unit. The transposed convolution operation is used to perform up-sampling in the decoding unit. The original U-Net utilizes cropping and copying feature maps to fuse coding unit information. U-Net has the following advantages: First, the U-Net embraces an extraordinary encoding and decoding unit, which can simultaneously get overall locations and context. Since most medical imaging is representative small sample datasets, U-Net can work with fewer training samples and achieve superior performance.

At present, many excellent medical image segmentation models are based on improvements made by U-Net. For instance, Tarek M et al. proposed R2U-Net [23], which improves U-Net by applying the recurrent residual convolutional block to train deeper networks. However, this model is liable to overfitting when training a small sample of medical datasets. Golnaz et al. proposed the Dropblock [24] module, which can effectively overcome the network overfitting. Guo et al. proposed structured Dropout U-Net (SD-UNet) [25], which adopts structured Dropblock instead of Dropout in the conventional convolutional layer to prevent overfitting. Although it can overcome overfitting, it does not adequately detect blood vessels when segmenting tiny blood vessels in fundus images. Wang et al. proposed DEU-Net [26], which significantly heightens the network’s performance by pixel-level prediction. It tends to ignore the tiny blood vessels during training. Guo et al. proposed spatial attention U-Net (SA-UNet) [27], which applies a spatial attention mechanism to concentrate on more valuable pixels and suppress background pixels to heighten the expressive capacity of the model, the segmentation effect of this network at the intersection of thick and thin blood vessels is not good.

To enhance the algorithm’s performance, the researchers mainly focused on the three elements of the network: depth, width, and cardinality. Except for these factors, “attention” has a powerful effect on the network’s performance. Woo, et al. proposed the convolutional block attention module (CBAM) [28], which connects different attention modules in series to learn what to emphasize or suppress. The CBAM module performed well in classification tasks. Fu et al. proposed the dual attention network (DANet) [29] to integrate local and global features adaptively to overcome the difficulty of capturing context information in computer vision tasks. Although the above attention mechanism models enhance the network’s performance, it makes the network model more complex and accompanied by increased parameters. Wang et al. proposed efficient channel attention (ECA-Net) [30] to achieve the trade-off between performance and complexity models. It reaches the local cross-channel information exchange without dimensionality reduction, which diminishes the complexity of the model whereas keeping up performance. The fundus image will be affected by uneven illumination and other factors during imaging, and the discontinuous characteristics of some small blood vessels, which will cause the blood vessel pixels not to be sufficiently detected by the model. Therefore, we proposed a structure containing attention mechanisms and a U-shaped structure, which can better locate and extract the tiny blood vessels in the fundus image.

## Methodology

This paper is devoted to proposing a valuable deep learning model to obtain a clear fundus blood vessel structure. Each pixel of fundus images is classified as a vessel (1) or background (0) pixel by the vessel segmentation model. Existing retinal vessel segmentation models are representative binary classification models.

**Fig 2:**
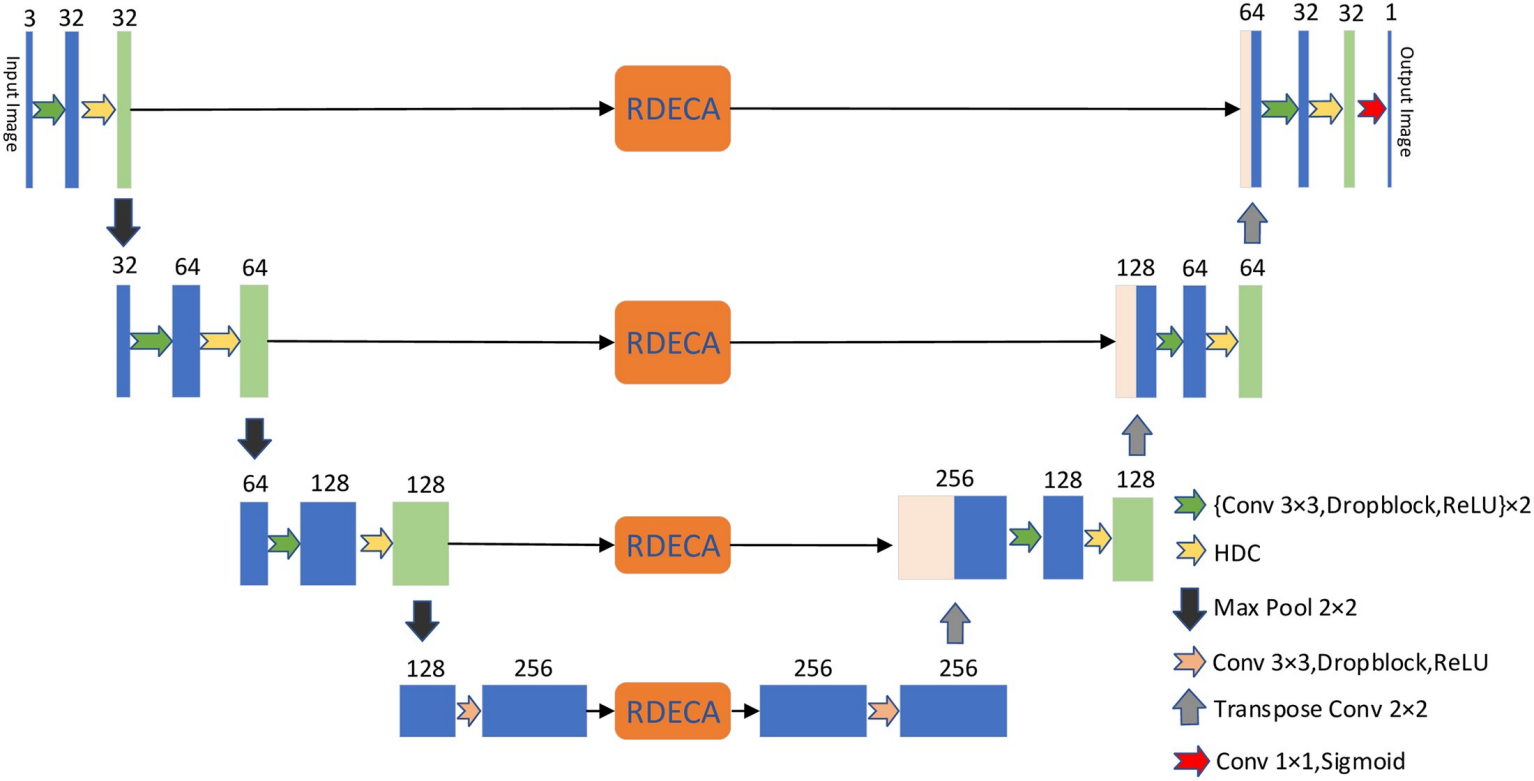
Diagrams of HDC-Net. The convolution operation extracts morphological and marginal information from the feature map (green arrow). The HDC module extracts retinal vessel features more fully in a hierarchical manner (yellow arrow). The RDECA module applied to skip connection can heighten the discriminative capacity of the model. Finally, a binary probability map was obtained by a 1×1 convolution operation and a sigmoid activation function (red arrow).

**Fig 3:**
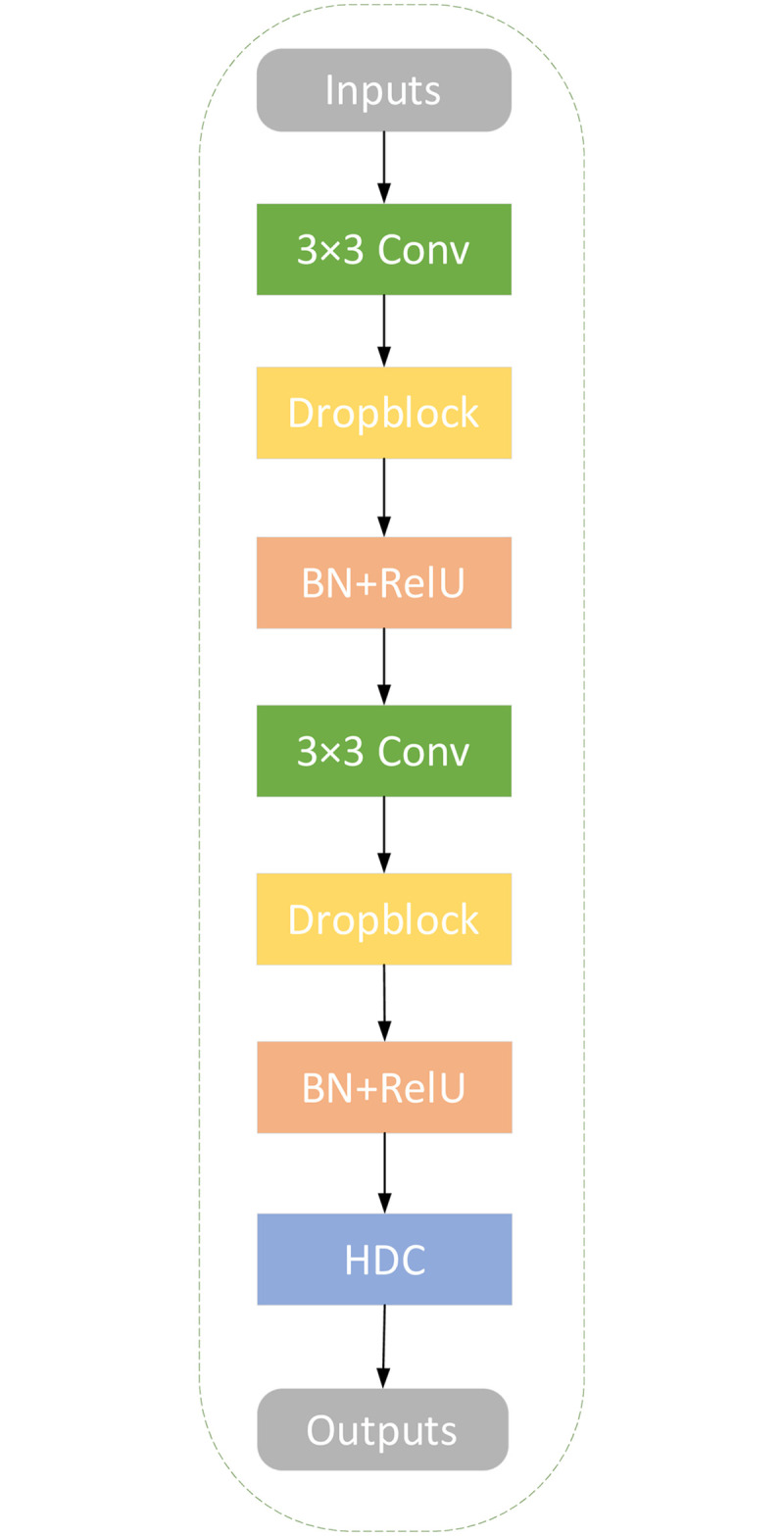
The operation flow in the encoding and decoding unit.

This section describes the structure of the HDC-Net for medical imaging analysis in detail. The HDC-Net architecture diagram is shown in Fig (2). We adopt SD-Net as the backbone network. SD-UNet can better overcome the problems caused by fewer samples in the training set. In the HDC-Net model, basic convolution operations are carried out in the encoding and decoding units, followed by the HDC module, to detect multi-scale vascular information in fundus images adequately. The operation flow of each layer in the encoding and the decoding unit are shown in Fig (3). Skip connection with the RDECA module can realize local cross-channel information exchange to improve the network’s ability to segment blood vessels.

### The Dropblock of regularization method

**Fig 4:**
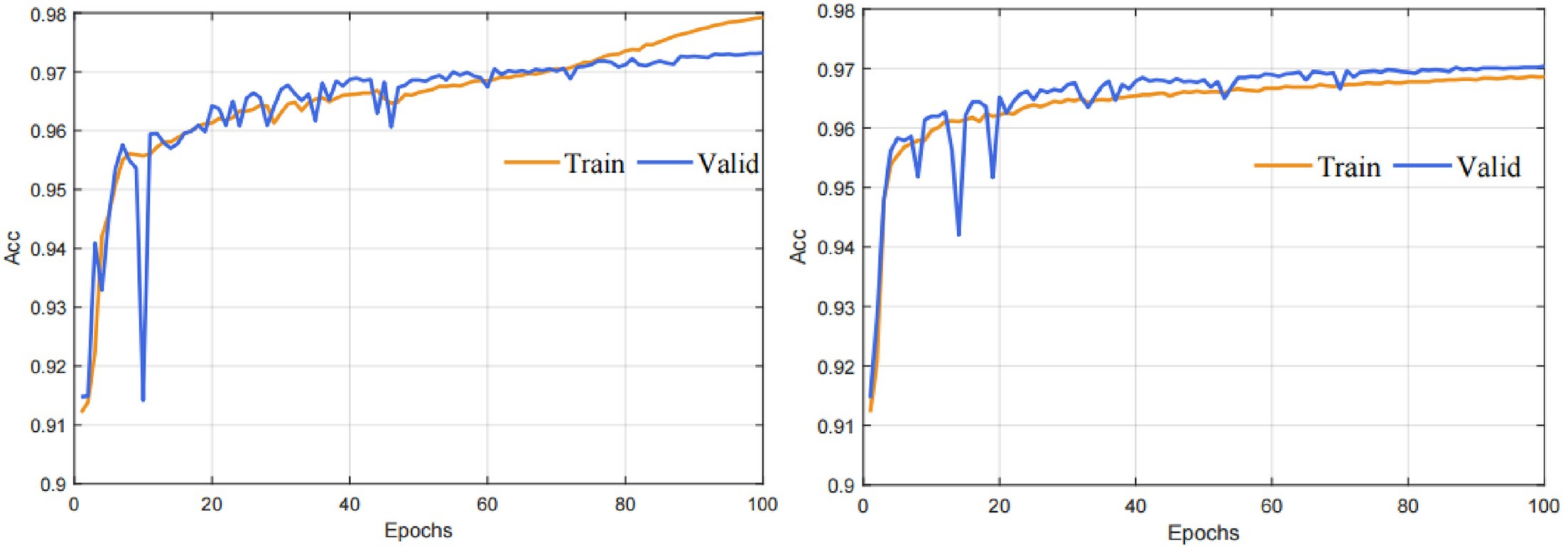
Comparison U-Net with HDC-Net models training 100 epochs on DRIVE dataset.

As we all know, marking the retinal blood vessels is laborious work, and the quantity of images is insufficient in most of the existing fundus datasets. Although the datasets have been augmented before inputting to the network, the network will still be overfitting during the training process. As shown in Fig (4) (left), When the training time reaches 80 epochs, the accuracy of the training set improves significantly while the validation set improves very slowly, it is an overfitting phenomenon. Dropblock is a structured form of Dropout that successfully avoids overfitting issues in our network. The distinction between Dropout and Dropblock is that Dropout randomly discards a single pixel, while Dropblock randomly discards a small pixel patch in the feature map. In addition, batch normalization (BN) and ReLU can significantly reduce the time required for network convergence in the basic convolution unit with Dropblock. The Dropblock module can perfectly solve overfitting in the HDC-Net. As shown in Fig (4) (right), The difference in accuracy between the training and validation sets is relatively stable over the overall training process.

### HDC module

Recent medical studies have shown the importance of high-quality segmentation of vascular structures for the early treatment of ophthalmic diseases. However, fundus images have many fragile vessels that are difficult to visualize with the naked eye and often overlooked by researchers. This section introduces the HDC module that allows for adequate detection and segmentation of retinal vessels.

**Fig 5:**
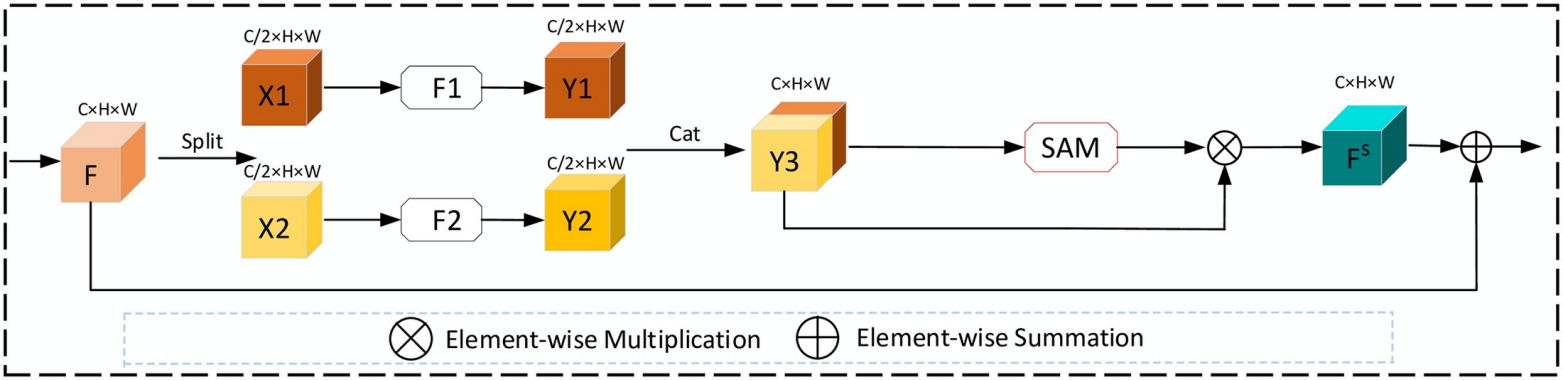
The operation flow of the HDC module. Among them, F1 and F2 represent dilated convolutions with dilation rates of 1 and 2, respectively.

The HDC module is a hierarchical structure, and it divides the input feature map into two parts along the channel axis. The feature conversion process takes place in these two parallel branches [31]. The feature maps generated by the two parallel branches are concatenated into a new feature map along the channel axis. In this case, each filter is responsible for a particular function in the HDC module. From the HDC module diagram in Fig (5), we can see that the channel number and resolution of the feature maps are unchanged between out and input features so that the HDC module can be used as a general module for fundus image segmentation tasks.

The input feature map (F) is divided evenly along the channel axis into two parts, denoted by X1 and X2, respectively. To effectively collect context information of each spatial position within the image, the convolution feature transformation is carried out in two spaces of different scales. The different receptive fields of the kernel can detect different scale information, and it can realize the comprehensive detection of blood vessels by the fusion of multi-scale structures. Dilated convolutions [32] with dilation rates of 1 and 2 were used to extract edge structure information of the retinal vessels, and Y1 and Y2 respectively represented the transformed feature maps. Dilated convolution changes the receptive field of the kernel to extract structural more fully and edge information of the vessels. The Y1 and Y2 concatenated along the channel axis to form a new feature map (Y3), and then the SAM was utilized for adaptive feature refinement. It is an approach that can detect neglected fragile blood vessels.

**Fig 6:**
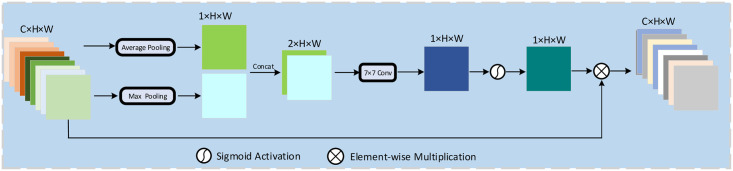
Diagram of the spatial attention in the HDC module.

In the SAM module, average-pooling can aggregate spatial information, while max-pooling can highlight different object features in an image. SAM models that contain two different pooling methods can infer more refined information, enhancing the network’s multi-scale perception capabilities and optimally capturing global key details. The operation flow of SAM is shown in Fig (6). The output *F*^*S*^ of the SAM module can be express as:

$$\begin{array}{c} F^{S}=\sigma (f^{7\times 7}\left(\text{cat}\left[\;\text{MaxPool}\;\left(Y1\right),\;\text{AveragePool}\;\left(Y2\right)\right]\right)) \end{array}$$ (1)

Where *f*^7×7^ means a convolution operation with a kernel size of 7, *σ*(⋅) represents the Sigmoid functions, and cat[⋅] presents the concatenate operation. In addition, the residual connection (RC) between the input and the output feature maps are utilized to prevent the overfitting and compensate for the loss of characteristic information to feature transformation.

### RDECA module

According to recent studies, it is common to apply attention mechanisms to deep learning models to heighten performance. However, most basic strategies are devoted to creating more complex attention modules to obtain superior performance, which unavoidably increases the difficulty of realization. Wang et al. proposed an ECA-Net, which adopts a 1D convolution operation to realize the information exchange between adjacent channels, significantly reducing the model’s parameters while keeping up with good performance. The ECA-Net only utilizes average-pooling to aggregate spatial information in feature maps, but max-pooling can gather more prominent information.

**Fig 7:**
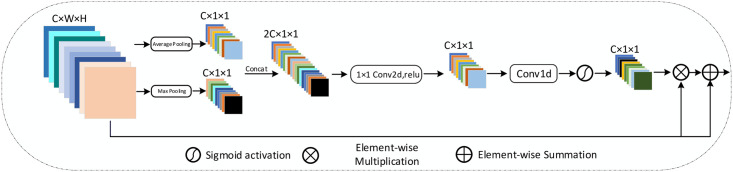
Diagram of RDECA. As shown in illustration, it uses both max-pooling and average-pooling to generate descriptors.

The RDECA module utilizes the max-pooling and average-pooling simultaneously to gather more abundant feature information, so it achieves accurate segmentation to some extent. The complete structure of the RDECA module is shown in Fig (7). The RDECA module utilizes different forms of pooling operations to generate different attention descriptors. The different channel attention descriptors are concatenated along the channel axis to retain more practical information than the element-wise summation. The 2D convolution with a kernel size of 1 is adopted to reduce the channels, followed by ReLU to activate the module. The 1D convolution is utilized to realize local cross-channel information exchange without the dimensionality reduction, and then the sigmoid function is adopted to generate the final channel attention descriptor.

**Fig 8:**
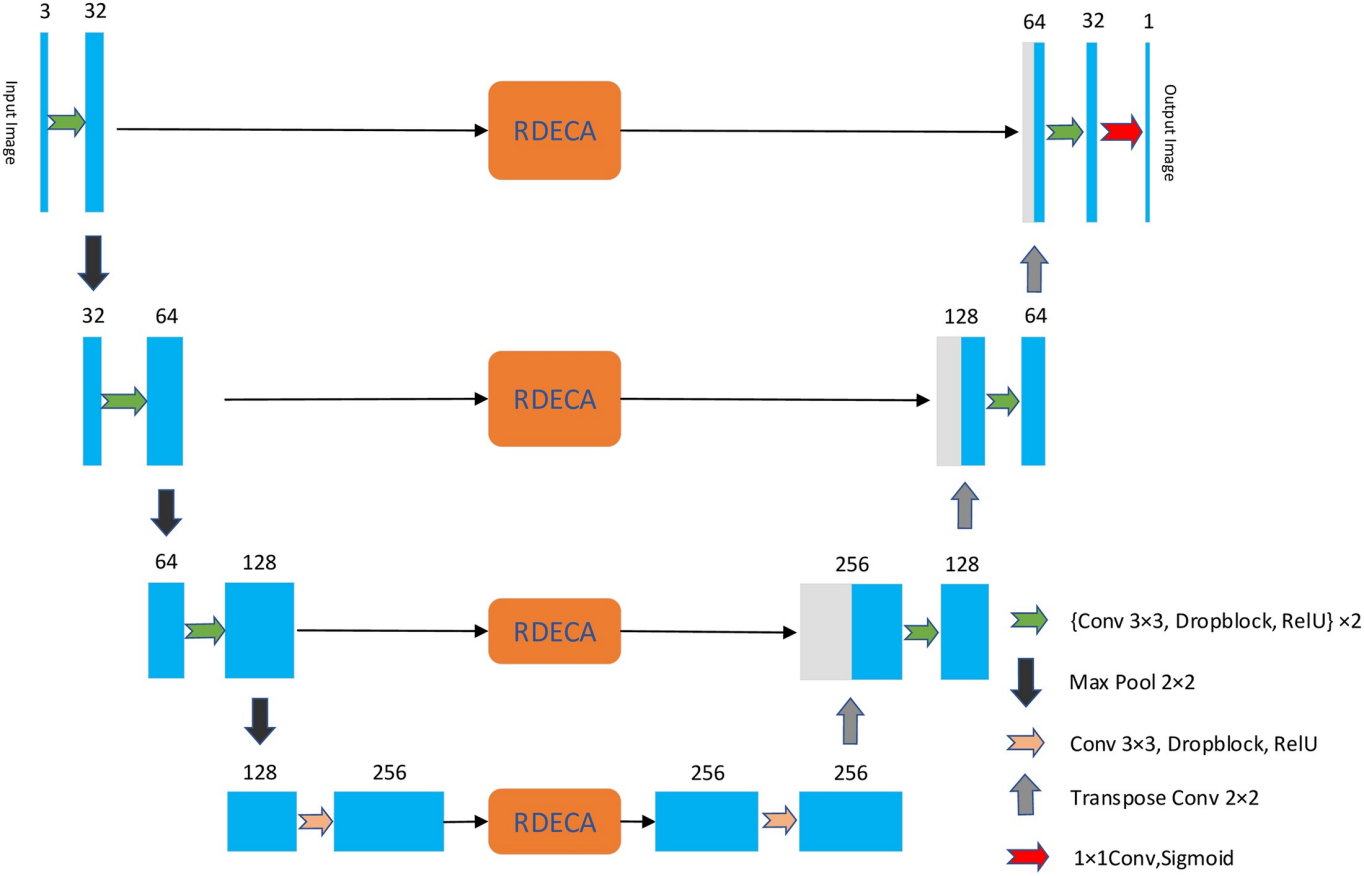
The diagram when the RDECA module is applied to SD-UNet.

Last but not least, the RC [33] is applied between the input and the final output to effectively prevent the overfitting caused by the network being too complex, and it also plays a role in supplementing information. In our experiment, the kernel size of 1D convolution is 3. In addition, Fig (8) shows the structure when the RDECA module is applied to SD-UNet only.

## Datasets and metrics

### The datasets

Although deep learning networks can effectively capture feature information from data that has not been pre-processed, they tend to perform better on pre-processed images. In addition, DRIVE [34], CHASE-DB1 [35] and STARE [36] are typical small sample datasets, so it is essential to pre-process the data before training. The DRIVE consists of 40 color images, which are from the Dutch diabetic retinopathy screening project. The CHASE-DB1 consists of 28 color fundus images derived from retinal imaging of 14 children. The STARE dataset consists of 20 fundus images, of which 10 have lesions, and 10 do not.

**Fig 9:**
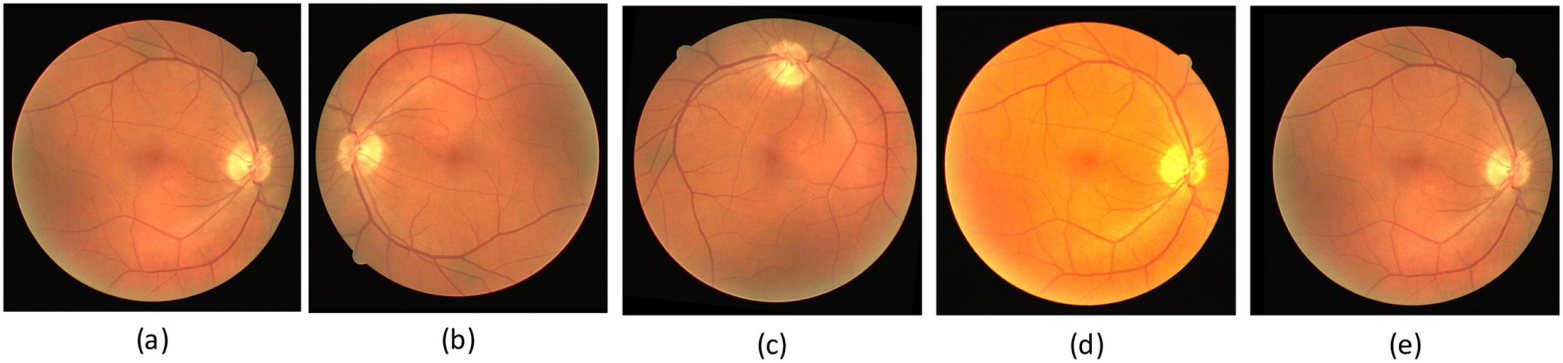
The four pre-processing methods for the DRIVE dataset. (a) Original image; (b) Image after flip; (c) Image after arbitrary angle rotation; (d) Image after adjust hue, brightness, contrast (e) Image after adding Gaussian noise.

**Fig 10:**
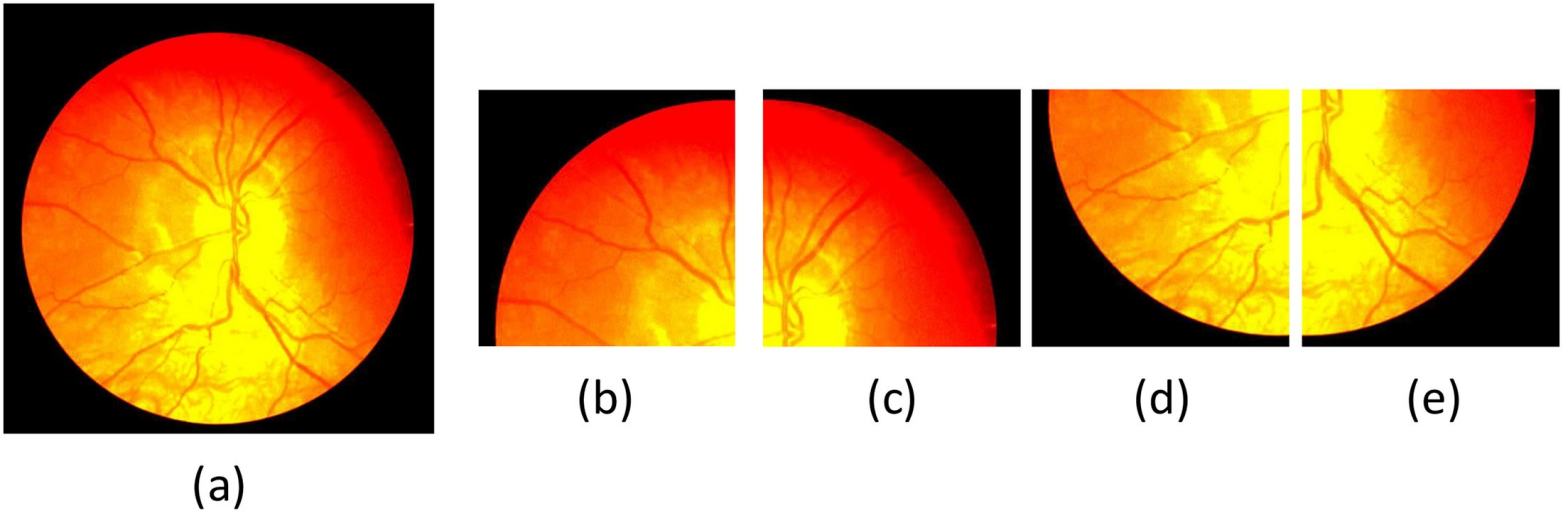
Images after processing by four different methods. (a) Image after pre-processing; (b) /(c) /(d) /(e) Images after crop operations.

The DRIVE dataset consists of 40 fundus images with a resolution of 584×565, of which training and test images each account for half. As the image’s resolution does not match the network, we change the image’s resolution by padding 0 pixels around the image. The resolutions of the DRIVE, CHASE-DB1, and STARE datasets were 565×584, 999×960, and 700×605. We adjusted the resolution of the images in the DRIVE, CHASE-DB1, and STARE datasets to 592×592, 1008×1008, and 704×704, respectively. The image resolutions were adjusted to be consistent with the original images in the three datasets during the evaluation process. In addition, we utilized four data augmentation methods: (1) random angle (0-360 degrees) rotation; (2) adding Gaussian noise; (3) adjust the hue, contrast, and brightness; (4) horizontal, vertical and diagonal flips; The images after each pre-processing step shown in Fig (9). In addition, the resolution of the image is too large for the network to train. Each image is cropped into four images with a resolution of 512×512 on the CHASE-DB1 dataset. The images after the cropping step are shown in Fig (10).

### The metrics

The output result of the HDC-Net is a probability prediction map, which describes the possibility of pixels as blood vessels. On the paper, the threshold is set as 0.5. If the predicted value of pixels in the probability map is greater than the threshold, it is considered a blood vessel pixel; otherwise, it is considered a background pixel. The probability maps compared with the corresponding ground truths, each element of the output image classified as True Positive (TP), False Positive (FP), True Negative (TN), and False Negative (FN). Sensitivity (SE) measures the proportion to which 1 pixel is predicted as blood vessels in the probability map. Specificity (SP) measures the proportion to which 0 pixels are predicted as background in the probability map. Accuracy (ACC) measures the proportion to which pixels are correctly predicted in the probability map. In addition, we also calculated the f1-score(F1) because it can better measure precision and recall at the same time.

$$\begin{array}{c} SE=\frac{TP}{TP+FN} \end{array}$$ (2)

$$\begin{array}{c} SP=\frac{TN}{TN+FP} \end{array}$$ (3)

$$\begin{array}{c} ACC=\frac{TN+TP}{TP+TN+FP+FN} \end{array}$$ (4)

$$\begin{array}{c} \;\text{Precision}=\frac{TP}{TP+FP} \end{array}$$ (5)

$$\begin{array}{c} \;\text{Recall}=\frac{TP}{TP+FN} \end{array}$$ (6)

$$\begin{array}{c} F1=2\times \frac{\;\text{Precision}\;\times \;\text{Recall}\;}{\;\text{Precision}\;+\;\text{Recall}\;} \end{array}$$ (7)

We also utilized the area under the curve (AUC) to evaluate our model to evaluate the network’s performance further. AUC is usually used to measure the performance of a binary classification model. If the AUC value is closer to 1, it means that the model’s performance is better.

## Results and analysis

### Implementation details

The HDC-Net model was evaluated on the DRIVE, CHASE-DB1, and STARE datasets, respectively. All models were trained from scratch on the training set and evaluated on the testing set. We use the Adam optimizer and a binary cross-entropy loss function to optimize our network. For the DRIVE dataset, we set the training epoch, learning rate, and batch size to 100, 0.008, and 2, respectively. For the CHASE-DB1 dataset, we set the training epoch, learning rate, and batch size to 50, 0.008, and 2, respectively. For the STARE dataset, we set the training epoch, learning rate, and batch size to 80, 0.008, and 2, respectively. In addition, for the Dropblock, we set the discard blocks and dropout rates to 7 and 0.15, respectively. The implementation is based on the public Pytorch, and all experiments run on Tesla V100-PCIE-16GB.

### Ablation experiment

The SD-UNet was selected serves as our baseline. Tables 1–3 show the results of SD-UNet, SD-UNet + RDECA, SD-UNet + HDC, and HDC-Net on the three datasets (DRIVE, CHASE-DB1, STARE) respectively. In addition, to prove that the RC in the RDECA module plays an essential role in our model, we also included SD-UNet+RDECA(no RC) and HDC-Net(no RC) in the ablation experiment. The ablation experiments show that the RDECA module was applied to the baseline, and the SP and ACC have increased by 0.02%/0.14%/0.09%, 0.04%/0.06%/0.02% on the three datasets, respectively. When the HDC module is applied to the baseline, the ACC, F1, and AUC of the SD-UNet+HDC increased by 0/0.11%/0.07%, 0.16%/0.88%/0.21%, and 0.03%/0.15%/0.12% on the three datasets, respectively, which shows our proposed HDC module can extract more vascular information.

**Table 1:** The ablation experiment based on DRIVE.

Model	SE	SP	ACC	F1	AUC
SD-UNet	0.7933	0.9855	0.9687	0.8157	0.9851
SD-UNet+RDECA(no RC)	0.7940	0.9854	0.9686	0.8153	0.9854
SD-UNet+RDECA	0.7956	**0.9857**	0.9691	0.8181	0.9857
SD-UNet+HDC	0.8002	0.9849	0.8687	0.8173	0.9854
HDC-Net(no RC)	0.8150	0.9833	0.9685	0.8189	0.9851
HDC-Net	**0.8258**	0.9829	**0.9692**	**0.8239**	**0.9871**

**Table 2:** The ablation experiment based on CHASE-DB1.

Model	SE	SP	ACC	F1	AUC
SD-UNet	0.8270	0.9836	0.9731	0.8039	0.9876
SD-UNet+RDECA(no RC)	0.8138	0.9848	0.9735	0.8023	0.9872
SD-UNet+RDECA	0.8151	0.9850	0.9737	0.8049	0.9875
SD-UNet+HDC	**0.8313**	0.9843	0.9742	0.8109	**0.9891**
HDC-Net(no RC)	0.8133	**0.9860**	0.9745	0.8096	0.9884
HDC-Net	0.8227	0.9853	**0.9745**	**0.8113**	0.9884

**Table 3:** The ablation experiment based on STARE.

Model	SE	SP	ACC	F1	AUC
SD-UNet	0.8291	0.9855	0.9735	0.8297	0.9900
SD-UNet+RDECA(no RC)	0.8290	0.9857	0.9736	0.8295	0.9905
SD-UNet+RDECA	0.8230	0.9864	0.9737	0.8267	0.9909
SD-UNet+HDC	0.8239	0.9867	0.9742	0.8318	0.9912
HDC-Net(no RC)	0.8168	**0.9881**	0.9748	0.8339	0.9912
HDC-Net	**0.8369**	0.9866	**0.9751**	**0.8385**	**0.9913**

Furthermore, the ablation experiments show that RDECA modules with an RC structure perform better than those without an RC structure. Therefore, the RC structure is conducive to improve the performance of the model. The segmentation performance of HDC-Net that combines the advantages of these two modules is better than applying the RDECA module or HDC module to the baseline alone.

**Fig 11:**
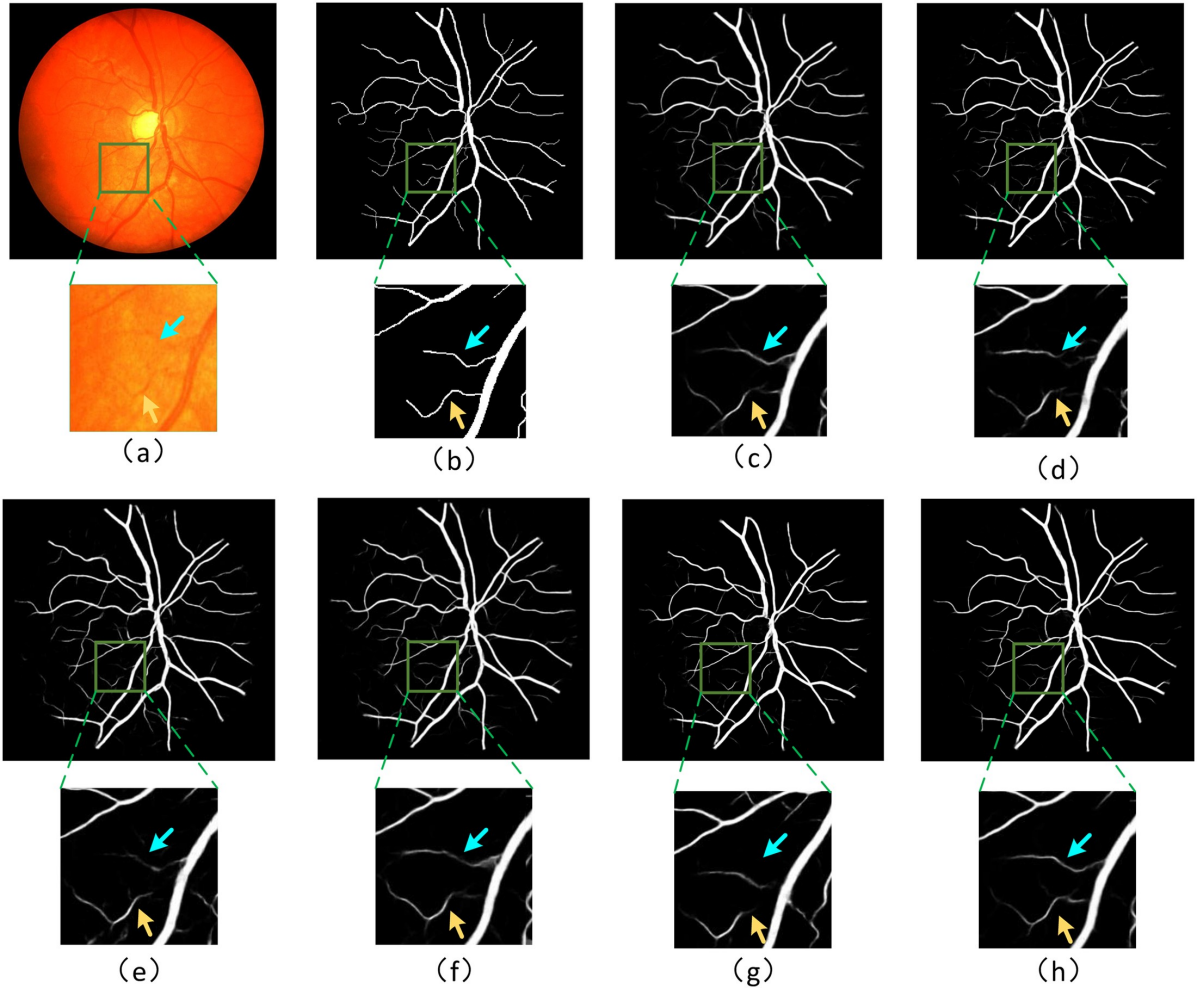
Enlarge the image for better observation; (a)Visualization image of test examples from the CHASE-DB1 dataset; (b)Corresponding **ground truth**; (c)Visualization results from **U-Net**; (d)Visualization image from **SD-UNet**; (e)Visualization image from **SD-UNet+RDECA**; (f)Visualization image from **SD-UNet+HDC**; (g)Visualization image from **SA-UNet**; (h)Visualization image from **HDC-Net(ours)**.

**Fig 12:**
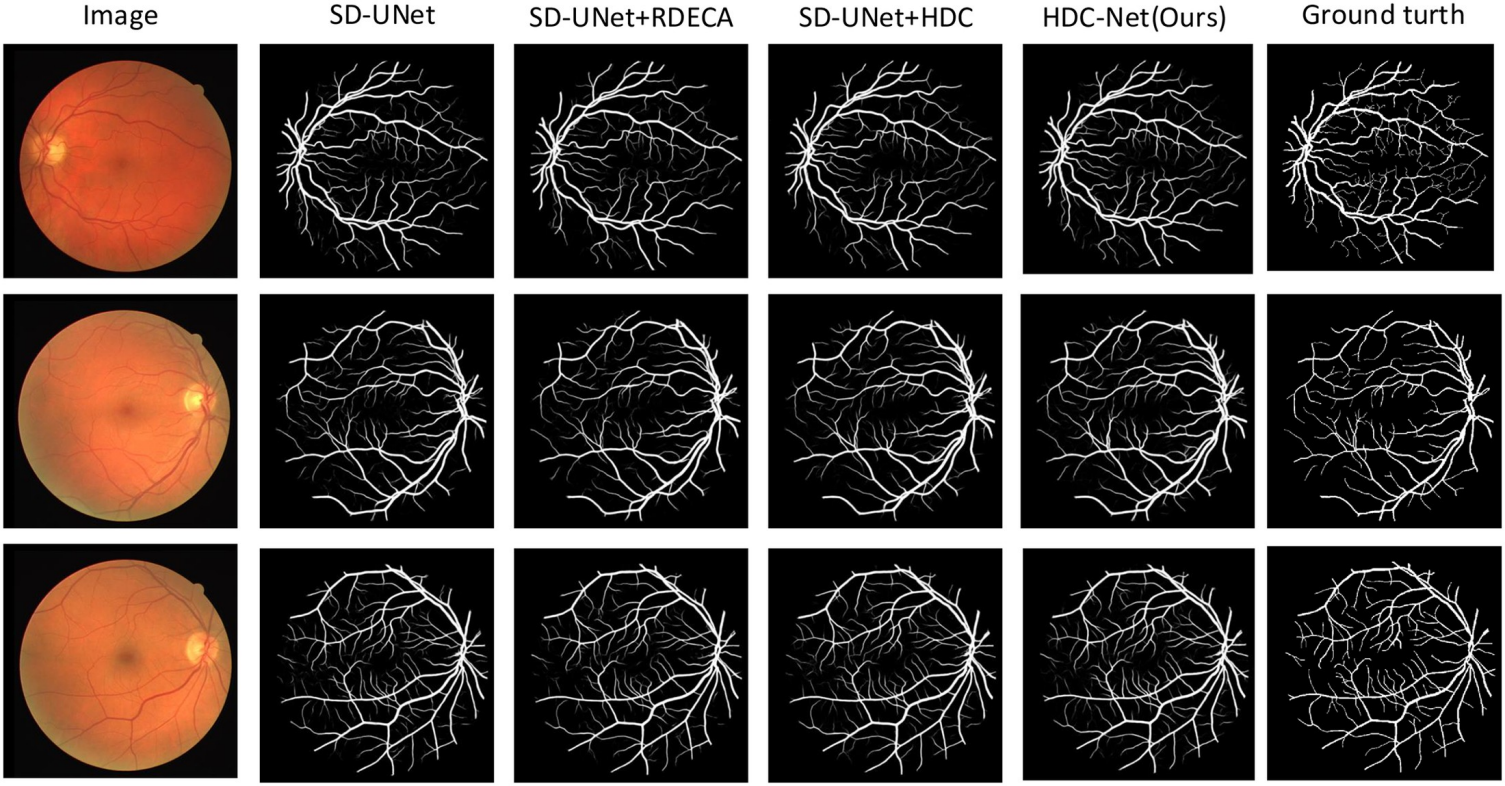
The visualization of the DRIVE dataset.

**Fig 13:**
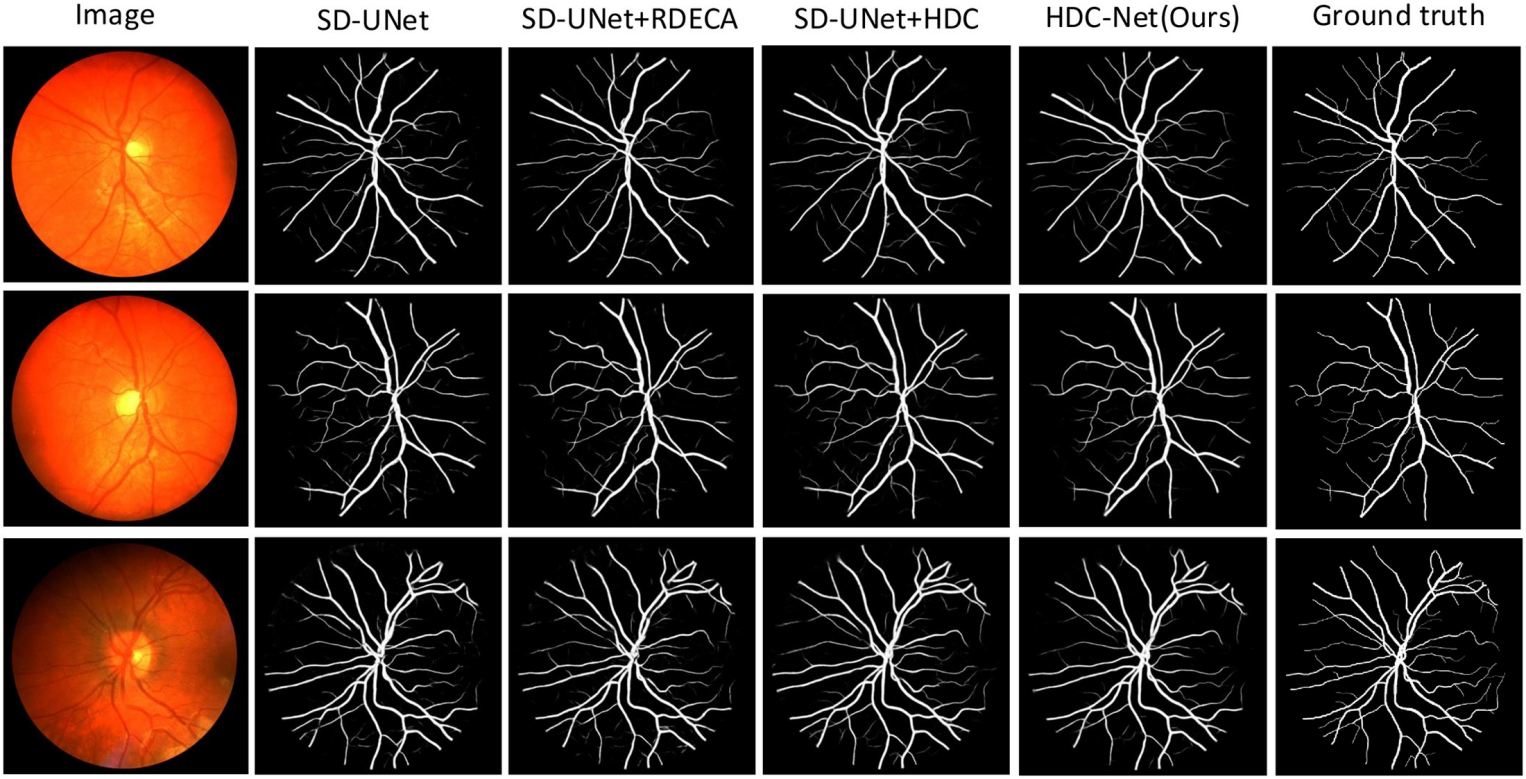
The visualization of the CHASE-DB1 dataset.

**Fig 14:**
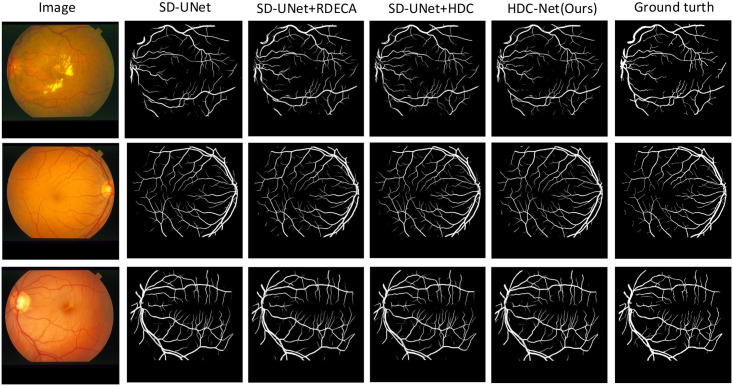
The visualization of the STARE dataset.

In Fig (11), we show the visualization image of the test example on the CHASE-DB1 dataset, including the segmentation results obtained by U-Net, SD-UNet, SD-UNet+RDECA, SD-UNet+HDC, SA-UNet, HDC-Net, and the corresponding ground truth. We know that the segmentation results obtained by SD-UNet are not accurate enough when segmenting small curved blood vessels from the visualization images. Although the segmentation results of SD-UNet + RDECA and SD-UNet + HDC are more accurate than SD-UNet, the edge structure of blood vessels is exceptionally rough and unsmooth. Compared with SD-UNet+RDECA and SD-UNet+HDC, the blood vessels segmented by SA-UNet perform better in terms of edge structure, but it performs poorly at the intersection between small and thick blood vessels. In short, the results of HDC-Net are the best from the perspective of indicators or visualizations, and it can ideally overcome the shortcomings of SA-UNet. Compared with the baseline, the result of HDC-Net has higher accuracy and can get a more precise edge structure. It demonstrates HDC-Net is effective for blood vessel segmentation. To further analyze the visualization images, we show more segmentation examples on DRIVE, CHASE-DB1, and STARE in Figs (12)–(14) respectively.

### Comparative experiment

To assess the effectiveness of HDC-Net, we compared the segmentation results of HDC-Net with other models applied to medical image segmentation. As shown in Table [4], HDC-Net reached to 0.8258, 0.9829, 0.9692, 0.8239, and 0.9871 for SE, SP, ACC, F1, and AUC, respectively on the DRIVE datasets, it shows that HDC-Net has outperformed than most other retinal vessel segmentation methods. From Table [5], we can see that compared with other advanced methods, the HDC-Net achieved the highest SP, ACC, and AUC, which are 0.9853, 0.9745, and 0.9884, respectively on the CHASE-DB1 dataset. Although the SE and F1 are not superior to other methods, they are also comparable to other methods. Table [6] shows the results of HDC-Net compared to other state-of-the-art methods. HDC-Net has the highest ACC, and other metrics are better than most other existing methods on the STARE dataset. In general, HDC-Net performs better than other existing methods when performing retinal vessel segmentation tasks. In the segmentation diagram, the segmented vessels are not only more precise but also have better continuity. The experimental results show that the HDC-Net algorithm with multi-scale awareness and enhanced discrimination capabilities performs well in the retinal vessel segmentation task and can detect and extract vessels adequately and accurately, which can be used for other retinal vessel segmentation tasks. In addition, we further compared the parameters of HDC-Net in relation to other models. As shown in Table [7], although HDC-Net does not have the fewest parameters, it has the best performance in retinal vessel segmentation, and it has significantly fewer parameters than R2-UNet.

**Table 4:** Results of HDC-Net and other methods on DRIVE dataset.

Model	SE	SP	ACC	F1	AUC
[18]	0.7884	0.9833	0.9662	0.8031	0.9812
[23]	0.7792	0.9813	0.9556	0.8171	0.9784
[27]	0.7850	**0.9860**	0.9684	0.8136	0.9829
[37]	0.7851	0.9724	0.9559	-	0.8787
[38]	0.7262	0.9803	0.9475	0.7786	-
[39]	0.7653	0.9818	0.9542	-	0.9752
[40]	**0.8432**	0.9813	0.9520	0.8163	-
[41]	0.8252	0.9787	0.9649	-	0.9780
[42]	0.8252	0.9764	0.9569	**0.8289**	0.9822
**HDC-Net(ours)**	0.8258	0.9829	**0.9692**	0.8239	**0.9871**

**Table 5:** Results of HDC-Net and other methods on CHASE-DB1 dataset.

Model	SE	SP	ACC	F1	AUC
[18]	0.8183	0.9838	0.9728	0.8000	0.9875
[23]	0.7756	0.9820	0.9634	0.7928	0.9815
[27]	0.8154	0.9847	0.9735	0.8028	0.9872
[37]	0.7776	0.9634	0.9505	-	0.8705
[39]	0.7633	0.9809	0.9610	-	0.9781
[41]	**0.8440**	0.9810	0.9722	-	0.9830
[42]	0.8199	0.9827	0.9665	**0.8280**	0.9865
**HDC-Net(ours)**	0.8227	**0.9853**	**0.9745**	0.8113	**0.9884**

**Table 6:** Results of HDC-Net and other methods on STARE dataset.

Model	SE	SP	ACC	F1	AUC
[18]	0.7955	0.9859	0.9710	0.8119	0.9887
[23]	0.8298	0.9862	0.9712	**0.8475**	**0.9914**
[27]	0.8167	**0.9876**	0.9744	0.8305	0.9906
[38]	0.7865	0.9730	0.9835	0.7750	-
[39]	0.7581	0.9846	0.9612	-	0.9801
[40]	**0.8630**	0.9730	0.9620	0.8233	-
[41]	0.8397	0.9792	0.9659	-	0.9810
**HDC-Net(ours)**	0.8369	0.9866	**0.9751**	0.8385	0.9913

**Table 7:** Parameter comparison of HDC-Net with other models.

Model	U-Net	SD-UNet	R2-UNet	SA-UNet	RCED-Net	HDC-Net
parameters	2143905	3029729	**39091393**	1054931	2359808	7156771

Generalization ability is an important basis for evaluating deep learning models, and it is very important in real applications. We adopt a cross-training approach to assess the generalization ability of HDC-Net. In Table [8], we compare the generalization ability of two existing methods with HDC-Net, it uses the DRIVE dataset to train the model and then evaluates it on the STARE dataset, and vice versa. Table [8] shows that except SP in all indicators have reached the highest for testing on STARE dataset, and reached the highest SP, ACC, AUC for testing on DRIVE dataset. In general, based on the data analysis, it can be known that the generalization ability of HDC-Net is the best.

**Table 8:** Performance comparison of cross-training.

Dateset	Models	SE	SP	ACC	F1	AUC
STARE(trained on DRIVE)	[39]	0.7211	**0.9840**	0.9569	-	0.9708
	[40]	-	-	0.9569	0.7866	-
	HDC-Net	**0.7843**	0.9838	**0.9685**	**0.7919**	**0.9779**
DRIVE(trained on STARE)	[39]	**0.7292**	0.9815	0.9494	-	0.9599
	[40]	-	-	0.9499	**0.7866**	-
	HDC-Net	0.7214	**0.9892**	**0.9656**	0.7852	**0.9713**

## Conclusion

High-quality fundus segmentation images are good for clinical diagnosis. We have developed a retinal vessel segmentation framework based on deep learning. The pre-processed retinal images were fed into the network for training, and then the trained model was further evaluated. In HDC-Net, the HDC module can detect vascular structure information of different scales, and the RDECA module in the skip connection part facilitates the information exchange between the encoding and decoding units. The proposed model we put forward was evaluated on three publicly available datasets (DRIVE, CHASE-DB1, STARE). The experimental results show that the performance achieved is comparable to or even better than that achieved by most of the existing state-of-the-art methods. Based on the analysis of ablation experiments on three different datasets (DRIVE, CHASE-DB1, STARE), the overall improvement in the performance of HDC-Net compared to baseline was significant. The ACC, F1, and AUC improved by {0.05%, 0.82%, 0.2%}, {0.41%, 0.74%, 0.08%}, {0.16%, 0.88%, 0.13%} respectively, and it demonstrated that the proposed HDC and RDECA module are helpful for retinal vessel segmentation. The proposed HDC-Net is effective and achievable. In addition, most retinal lesions remain some similar symptoms, such as microaneurysms, hemorrhages, exudates, and other abnormalities found in the retina, so the proposed HDC-Net we put forward can be used as a general network to perform other retinal vascular segmentation tasks competently.

## References

[1] Smart TJ, Richards CJ, Bhatnagar R, Pavesio C, Agrawal R, Jones PH. A study of red blood cell deformability in diabetic retinopathy using optical tweezers. In: Optical trapping and optical micromanipulation XII. vol. 9548; 2015. pp. 954825. doi: 10.1117/12.2191281

[2] Winder RJ, Morrow PJ, McRitchie IN, Bailie JR, Hart PM. Algorithms for digital image processing in diabetic retinopathy. Comput Medical Imaging Graph. 2009;33(8):608–622. doi: 10.1016/j.compmedimag.2009.06.003 19616920

[3] Ikram MK, Witteman JC, Vingerling JR, Breteler MM, Hofman A, de Jong PT. Retinal vessel diameters and risk of hypertension: the Rotterdam Study. hypertension. 2006;47(2):189–194. doi: 10.1161/01.HYP.0000199104.61945.33 16380526

[4] Mendonca AM, Campilho A. Segmentation of retinal blood vessels by combining the detection of centerlines and morphological reconstruction. IEEE Transactions on Medical Imaging. 2006;25(9):1200–1213. doi: 10.1109/TMI.2006.879955 16967805

[5] Chaudhuri S, Chatterjee S, Katz N, Nelson M, Goldbaum M. Detection of blood vessels in retinal images using two-dimensional matched filters. IEEE Transactions on Medical Imaging. 1989;8(3):263–269. doi: 10.1109/42.34715 18230524

[6] Sofka M, Stewart CV. Retinal Vessel Centerline Extraction Using Multiscale Matched Filters, Confidence and Edge Measures. IEEE Transactions on Medical Imaging. 2006;25(12):1531–1546. doi: 10.1109/TMI.2006.884190 17167990

[7] Odstrcilík J, Kolár R, Budai A, Hornegger J, Jan J, Gazárek J, et al. Retinal vessel segmentation by improved matched filtering: evaluation on a new high-resolution fundus image database. IET Image Process. 2013;7(4):373–383. doi: 10.1049/iet-ipr.2012.0455

[8] Palomera-Pérez MA, Martinez-Perez ME, Benítez-Pérez H, Ortega-Arjona JL. Parallel Multiscale Feature Extraction and Region Growing: Application in Retinal Blood Vessel Detection. IEEE Transactions on Information Technology in Biomedicine. 2010;14(2):500–506. doi: 10.1109/TITB.2009.2036604 20007040

[9] Zhao YQ, Wang X, Wang X, Shih FY. Retinal vessels segmentation based on level set and region growing. Pattern Recognit. 2014;47(7):2437–2446. doi: 10.1016/j.patcog.2014.01.006

[10] Miri MS, Mahloojifar A. Retinal Image Analysis Using Curvelet Transform and Multistructure Elements Morphology by Reconstruction. IEEE Transactions on Biomedical Engineering. 2011;58(5):1183–1192. doi: 10.1109/TBME.2010.2097599 21147592

[11] Espona L, Carreira MJ, Penedo MG, Ortega M. Retinal vessel tree segmentation using a deformable contour model. In: 2008 19th International Conference on Pattern Recognition; 2008. pp. 1–4.

[12] Al-Diri B, Hunter A, Steel D. An Active Contour Model for Segmenting and Measuring Retinal Vessels. IEEE Transactions on Medical Imaging. 2009;28(9):1488–1497. doi: 10.1109/TMI.2009.2017941 19336294

[13] Zhao Y, Rada L, Chen K, Harding SP, Zheng Y. Automated Vessel Segmentation Using Infinite Perimeter Active Contour Model with Hybrid Region Information with Application to Retinal Images. IEEE Transactions on Medical Imaging. 2015;34(9):1797–1807. doi: 10.1109/TMI.2015.2409024 25769147

[14] Delibasis KK, Kechriniotis AI, Tsonos C, Assimakis ND. Automatic model-based tracing algorithm for vessel segmentation and diameter estimation. Comput Methods Programs Biomed. 2010;100(2):108–122. doi: 10.1016/j.cmpb.2010.03.004 20363522

[15] Zhang J, Li H, Nie Q, Cheng L. A retinal vessel boundary tracking method based on Bayesian theory and multi-scale line detection. Comput Medical Imaging Graph. 2014;38(6):517–525. doi: 10.1016/j.compmedimag.2014.05.010 24974011

[16] Badrinarayanan V, Kendall A, Cipolla R. SegNet: A Deep Convolutional Encoder-Decoder Architecture for Image Segmentation. IEEE Transactions on Pattern Analysis and Machine Intelligence. 2017;39(12):2481–2495. doi: 10.1109/TPAMI.2016.2644615 28060704

[17] Krizhevsky A, Sutskever I, Hinton GE. ImageNet Classification with Deep Convolutional Neural Networks. In: Advances in Neural Information Processing Systems 25: 26th Annual Conference on Neural Information Processing Systems 2012. Proceedings of a meeting held December 3-6, 2012, Lake Tahoe, Nevada, United States; 2012. pp. 1106–1114.

[18] Ronneberger O, Fischer P, Brox T. U-Net: Convolutional Networks for Biomedical Image Segmentation. In: Medical Image Computing and Computer-Assisted Intervention—MICCAI 2015—18th International Conference Munich, Germany, October 5–9, 2015, Proceedings, Part III. vol. 9351 of Lecture Notes in Computer Science. Springer; 2015. pp. 234–241.

[19] Long J, Shelhamer E, Darrell T. Fully convolutional networks for semantic segmentation. In: 2015 IEEE Conference on Computer Vision and Pattern Recognition (CVPR); 2015. pp. 3431–3440. 10.1109/TPAMI.2016.2572683 27244717

[20] Dasgupta A, Singh S. A fully convolutional neural network based structured prediction approach towards the retinal vessel segmentation. In: 2017 IEEE 14th International Symposium on Biomedical Imaging (ISBI 2017); 2017. pp. 248–251.

[21] Lidong H, Wei Z, Jun W, Sun Z. Combination of contrast limited adaptive histogram equalisation and discrete wavelet transform for image enhancement. IET Image Process. 2015;9(10):908–915. doi: 10.1049/iet-ipr.2015.0150

[22] Aurangzeb K, Aslam S, Alhussein M, Naqvi RA, Arsalan M, Haider SI. Contrast Enhancement of Fundus Images by Employing Modified PSO for Improving the Performance of Deep Learning Models. IEEE Access. 2021;9:47930–47945. doi: 10.1109/ACCESS.2021.3068477

[23] Alom MZ, Hasan M, Yakopcic C, Taha TM, Asari VK. Recurrent Residual Convolutional Neural Network based on U-Net (R2U-Net) for Medical Image Segmentation. CoRR. 2018;abs/1802.06955.

[24] Ghiasi G, Lin T, Le QV. DropBlock: A regularization method for convolutional networks. In: Advances in Neural Information Processing Systems 31: Annual Conference on Neural Information Processing Systems 2018, NeurIPS 2018, December 3-8, 2018, Montréal, Canada; 2018. pp. 10750–10760.

[25] Guo C, Szemenyei M, Pei Y, Yi Y, Zhou W. SD-Unet: A Structured Dropout U-Net for Retinal Vessel Segmentation. In: 2019 IEEE 19th International Conference on Bioinformatics and Bioengineering (BIBE); 2019. pp. 439–444.

[26] Wang B, Qiu S, He H. Dual Encoding U-Net for Retinal Vessel Segmentation. In: Medical Image Computing and Computer Assisted Intervention—MICCAI 2019—22nd International Conference, Shenzhen, China, October 13-17, 2019, Proceedings, Part I. vol. 11764 of Lecture Notes in Computer Science. Springer; 2019. pp. 84–92.

[27] Guo C, Szemenyei M, Yi Y, Wang W, Chen B, Fan C. SA-UNet: Spatial Attention U-Net for Retinal Vessel Segmentation. In: 2020 25th International Conference on Pattern Recognition (ICPR); 2021. pp. 1236–1242.

[28] Woo S, Park J, Lee J, Kweon IS. CBAM: Convolutional Block Attention Module. In: Computer Vision—ECCV 2018—15th European Conference, Munich, Germany, September 8-14, 2018, Proceedings, Part VII. vol. 11211 of Lecture Notes in Computer Science. Springer; 2018. pp. 3–19.

[29] Fu J, Liu J, Tian H, Li Y, Bao Y, Fang Z, et al. Dual Attention Network for Scene Segmentation. In: 2019 IEEE/CVF Conference on Computer Vision and Pattern Recognition (CVPR); 2019. pp. 3141–3149.

[30] Wang Q, Wu B, Zhu P, Li P, Zuo W, Hu Q. ECA-Net: Efficient Channel Attention for Deep Convolutional Neural Networks. In: 2020 IEEE/CVF Conference on Computer Vision and Pattern Recognition (CVPR); 2020. pp. 11531–11539.

[31] Liu JJ, Hou Q, Cheng MM, Wang C, Feng J. Improving Convolutional Networks With Self-Calibrated Convolutions. In: 2020 IEEE/CVF Conference on Computer Vision and Pattern Recognition (CVPR); 2020. pp. 10093–10102.

[32] Yu F, Koltun V. Multi-Scale Context Aggregation by Dilated Convolutions. In: Bengio Y, LeCun Y, editors. 4th International Conference on Learning Representations, ICLR 2016, San Juan, Puerto Rico, May 2-4, 2016, Conference Track Proceedings; 2016.

[33] He K, Zhang X, Ren S, Sun J. Deep Residual Learning for Image Recognition. In: 2016 IEEE Conference on Computer Vision and Pattern Recognition (CVPR); 2016. pp. 770–778.

[34] Staal J, Abramoff MD, Niemeijer M, Viergever MA, van Ginneken B. Ridge-based vessel segmentation in color images of the retina. IEEE Transactions on Medical Imaging. 2004;23(4):501–509. doi: 10.1109/TMI.2004.825627 15084075

[35] Fraz MM, Remagnino P, Hoppe A, Uyyanonvara B, Rudnicka AR, Owen CG, et al. An Ensemble Classification-Based Approach Applied to Retinal Blood Vessel Segmentation. IEEE Transactions on Biomedical Engineering. 2012;59(9):2538–2548. doi: 10.1109/TBME.2012.2205687 22736688

[36] Hoover AD, Kouznetsova V, Goldbaum M. Locating blood vessels in retinal images by piecewise threshold probing of a matched filter response. IEEE Transactions on Medical Imaging. 2000;19(3):203–210. doi: 10.1109/42.845178 10875704

[37] Alhussein M, Aurangzeb K, Haider SI. An Unsupervised Retinal Vessel Segmentation Using Hessian and Intensity Based Approach. IEEE Access. 2020;8:165056–165070. doi: 10.1109/ACCESS.2020.3022943

[38] Zhou C, Zhang X, Chen H. A new robust method for blood vessel segmentation in retinal fundus images based on weighted line detector and hidden Markov model. Computer Methods and Programs in Biomedicine. 2020;187:105231. doi: 10.1016/j.cmpb.2019.105231 31786454

[39] Yan Z, Yang X, Cheng K. Joint Segment-Level and Pixel-Wise Losses for Deep Learning Based Retinal Vessel Segmentation. IEEE Trans Biomed Eng. 2018;65(9):1912–1923. doi: 10.1109/TBME.2018.2828137 29993396

[40] Zhuo Z, Huang J, Lu K, Pan D, Feng S. A size-invariant convolutional network with dense connectivity applied to retinal vessel segmentation measured by a unique index. Comput Methods Programs Biomed. 2020;196:105508. doi: 10.1016/j.cmpb.2020.105508 32563893

[41] Khan TM, Alhussein M, Aurangzeb K, Arsalan M, Naqvi SS, Nawaz SJ. Residual Connection-Based Encoder Decoder Network (RCED-Net) for Retinal Vessel Segmentation. IEEE Access. 2020;8:131257–131272. doi: 10.1109/ACCESS.2020.3008899

[42] Hu J, Wang H, Wang J, Wang Y, He F, Zhang J. SA-Net: A scale-attention network for medical image segmentation. PloS one. 2021;16:e0247388. doi: 10.1371/journal.pone.0247388 33852577 PMC8046243

